# ADP-Ribosylation Post-Translational Modification: An Overview with a Focus on RNA Biology and New Pharmacological Perspectives

**DOI:** 10.3390/biom12030443

**Published:** 2022-03-13

**Authors:** Giuseppe Manco, Giuseppina Lacerra, Elena Porzio, Giuliana Catara

**Affiliations:** 1 Institute of Biochemistry and Cell Biology, National Research Council of Italy, Via P. Castellino 111, 80131 Naples, Italy; elena.porzio@cnr.it; 2 Institute of Genetics and Biophysics “Adriano Buzzati-Traverso”, National Research Council of Italy, Via P. Castellino 111, 80131 Naples, Italy; giuseppina.lacerra@igb.cnr.it

**Keywords:** ADP-ribosylation, ARTs, mRNA regulation, MARylation, PARylation, poly-ADP-ribosylpolymerases (PARPs)

## Abstract

Cellular functions are regulated through the gene expression program by the transcription of new messenger RNAs (mRNAs), alternative RNA splicing, and protein synthesis. To this end, the post-translational modifications (PTMs) of proteins add another layer of complexity, creating a continuously fine-tuned regulatory network. ADP-ribosylation (ADPr) is an ancient reversible modification of cellular macromolecules, regulating a multitude of key functional processes as diverse as DNA damage repair (DDR), transcriptional regulation, intracellular transport, immune and stress responses, and cell survival. Additionally, due to the emerging role of ADP-ribosylation in pathological processes, ADP-ribosyltransferases (ARTs), the enzymes involved in ADPr, are attracting growing interest as new drug targets. In this review, an overview of human ARTs and their related biological functions is provided, mainly focusing on the regulation of ADP-ribosyltransferase Diphtheria toxin-like enzymes (ARTD)-dependent RNA functions. Finally, in order to unravel novel gene functional relationships, we propose the analysis of an inventory of human gene clusters, including *ARTDs*, which share conserved sequences at 3′ untranslated regions (UTRs).

## 1. Introduction

The activation of gene expression programs serves the cell to ensure coordinated cellular processes and fate resolutions [1]. This coordination can be achieved both at the transcriptional and post-transcriptional levels. Indeed, the transcription of new mRNA molecules, alternative mRNA splicing processes, mRNA stabilization/destabilization, as well as *de novo* protein synthesis are regularly switched on/off by the cell in response to intracellular/extracellular *stimuli*. In addition to this, the PTMs of proteins have been established to play a pivotal role in mediating cell response upon specific inputs, thus contributing to the arrangement of a continuously fine-tuned regulatory network [2]. As a result, post-translationally modified proteins can turn on/off their activity state, change cellular localization, turnover, and establish new protein–protein interactions [2]. The improvement of mass spectrometry (MS) technologies has largely increased the number of identified PTMs, which now accounts for around 400 [3], including acetylation, propionylation, methylation, phosphorylation, sumoylation, and ubiquitination, which have been proven to be involved in the regulation of cellular processes at diverse subcellular compartments. Of note, a growing body of literature has also outlined the interplay between the different PTMs [4,5,6], suggesting that the crosstalk is compelling for the right fulfillment of such diverse cell functions as, for example, gene expression, genome organization, cell division and DNA damage response. Given the fundamental roles exerted by PTMs, the discovery of specific inhibitors to address therapeutic treatments of diseases including cancer has greatly expanded in the last decade [7]. Likewise, the implementation of bioinformatic predictive tools has paved the way to the discovery of alternative drug targets for therapeutic intervention [8].

ADP-ribosylation is a reversible phylogenetically ancient mechanism of protein modification where the ADP-ribose moiety is covalently transferred from β-nicotinamide adenine dinucleotide (NAD^+^) onto target proteins with the release of nicotinamide [9,10].

In addition to proteins, ADPr reaction is responsible for the chemical modification of different targets as small molecules and nucleic acids, with a broad spectrum of effects in living organisms [11,12,13,14,15].

ADP-ribose transfer reactions are catalyzed by at least three evolutionarily unrelated superfamilies of enzymes, namely the ADP-ribosyltransferases, the Sirtuins, and the less studied TM1506 superfamily [10,16,17,18]. The TM1506 superfamily contains ADP-ribosylating enzymes only found in bacteria, such as the prototype protein from *Thermotoga maritima*, showing a deaminase-like fold that displays auto-ADP-ribosylation onto aspartate residue [18].

ADPr systems have been reported in all domains of life [11,19,20] including Archaea [21], Bacteria [22,23,24], viruses ([23] and the references therein), and the large part of Eukarya [9,10,25,26].

In this paper, after a brief review on ARTs distribution in the three domains of life, we refer to ADPr reactions in humans with a focus on the most recent findings in RNA biology, suggestive of new biological functions in physiological processes. Likewise, the dysregulation of some of the ADPr enzymes is also causative of pathological mechanisms, thus providing evidence of the need of an efficient gene expression regulatory network. To this end, we also consider the post-translational gene expression mechanism, known as “RNA operon”, as a putative regulatory system affecting mRNA transcripts that encloses those coding for ART enzymes as well. Therefore, the evaluation of novel therapeutic pharmacological opportunities targeting the ADPr reaction is also suggested.

## 2. ADP-Ribosyltransferase Activity during Evolution in the Three Domains of Life

ARTs represent one of the superfamilies of enzymes catalyzing ADP-ribosylation. They are ubiquitous, conserved throughout evolution and common to all three domains of life: Archaea, Bacteria and Eukarya. In Bacteria, ADP-ribosylation modification regulates a high number of cellular processes, including cell homeostasis as well as bacterial and viral pathogenic mechanisms, to cite the most known [27,28,29,30,31,32,33].

ADPr was originally discovered as a post-translational protein modification catalyzed by Diphtheria Toxin (DT) and Cholera Toxin (CT). Both of them harbor an ART catalytic domain showing very limited sequence similarity [34,35], despite a conserved structural organization of the core fold, as found in all members of the ART superfamily [12,13,14,15,34,35,36].

ARTs fall into two major subclasses, distinguished based on conserved structural motifs in the catalytic triad: H-Y-E-containing enzymes in the ADP-ribosyltransferase Diphtheria toxin-like (ARTD) subfamily, formerly known as poly-ADP-ribosylpolymerases (PARPs); and R-S-E-containing enzymes in the ADP-ribosyltransferase Clostridium toxin-like (ARTC) subfamily (related to C2 and C3 Clostridial Toxins).

Recently, by reconstructing the evolutionary history for the different members of ART superfamily, ARTs have been further divided in three major higher order clades where the most primordial branching clade results to be the H-H-h clade followed by a crown group comprised of the H-Y-[EDQ] and the R-S-E clades [11,12,13]. The H-H-h and H-Y-[EDQ] include the ARTD proteins, while the R-S-E clade includes the ARTC proteins, in the previously presented nomenclature for ART domains [12,13,14,15,34,35,36]. Thus, the presence of an active H residue in the first branch appears to be the ancestral feature of the ART superfamily, with the R in the R-S-E clade being a derived feature [11].

In Prokaryotes, according to Perina and colleagues (2014) [19], 28 PARP homologues can be found in 27 bacterial species, belonging to 6 different phyla of the Bacteria domain; therefore, this suggests a horizontal PARP gene transfer in Bacteria. Only one bacterium, *Microscilla marina*, possesses two PARP genes in its genome. Most of bacterial PARPs contain only two domains (WGR and PARP domains) and are most similar to the DNA repair-specific Clade 1 PARP homologues. The catalytic triad H-Y-E essential for the poly-ADP-ribosylation activity of ARTDs is conserved in the largest part (27 out of 28) of analyzed bacterial PARPs. In the remaining PARPs, only the third amino acid residue of the catalytic triad is conserved. This protein is most similar to PARP1 homologues, indicating that the mutations, which may have changed its ADPr function (poly- *vs* mono-ADP-ribosylation), occurred after horizontal gene transfer [19].

Despite the fact that ARTD genes have not been found in archaeal genomes, endogenous ADPr activity has been detected in the archaeon *Saccharolobus solfataricus*. The PARP-like enzyme from *S. solfataricus* (PARPSso) has been partially purified, possessing an oligo-ADP-ribosyltransferase activity with non-specific DNA binding activity [21]. The thermozyme undergoes auto-modification and modifies as target protein the 7-kDa protein (Sso7), an histone-like protein in sulphur-dependent extremophiles [37]. PARPSso is not structurally related to the known ARTDs [38] and localizes at the edge of the sulfolobal cell membrane [39]. Structurally, PARPSso belongs to the family of DING proteins, and similarly to other members of the DING family, it is a monomer of 40 kDa and has a single highly conserved phosphate-binding site that is involved in its phosphatase activity as other DING proteins from different organisms [40].

Recently, PARPSso has been reported as a multifunctional enzyme also displaying ATPase and low phosphatase activities in addition to the ability to ADP-ribosylate substrates [41]. The presence of multiple activities suggests that ADPr activity has evolved throughout the evolution up to humans, from promiscuous enzymes, as described for PARPSso [42]. However, even though genes encoding poly-ADP-ribosylglycohydrolases (PARG) enzymes have not been found, many archaeal genomes have been shown to encode other macrodomain-containing proteins. To date, Af1521 from *Archaeoglobus fulgidus* remains the best characterized archaeal macrodomain protein [43] capable of binding both ADP-ribose and PAR, displaying hydrolyzing activity onto mono-ADP-ribosylated substrates [44,45].

PARP genes have also been identified in four genomes of dsDNA viruses [19]. Most likely, the genes have been gained from their hosts. The catalytic triad H-Y-E is fully conserved in three of these viral PARPs, whereas only one has replaced the E residue with D, which may suggest that these enzymes are active ARTs. Some viruses use PAR metabolism for their replication. For example, the Herpes Simplex Virus and Epstein–Barr Virus require PARP activity for efficient replication [46,47].

Other examples of novel ADPr players are provided by the viral macro domains, present in many dangerous viruses, including the SARS and SARS-CoV-2 coronaviruses [48], and by the recently characterized novel ART-like domain (DUF3715) in the human transgene activation suppressor (TASOR) protein involved in gene silencing [49,50,51]. However, it is not clear whether members of the ART superfamily were present in the Last Universal Common Ancestor (LUCA) or whether they firstly arose in Bacteria [11]. It is possible that starting from an ancestral version the ART superfamily diverged rapidly in terms of sequence, structure and active site residues, recognizing a wide range of substrates in the bacterial super-kingdom [11]. Probably, ARTs evolving in these systems have been repeatedly acquired by lateral transfer throughout eukaryotic evolution.

## 3. Human Proteins in ADPr Biology

### 3.1. ADP-Ribosyltransferases

ADPr reaction in humans is mainly catalyzed by members of the ART superfamily, including both ARTD and ARTC enzymes [12,36]. Although in this paper we will report only about ARTs, a few members of the Sirtuins (SIRTs) a structurally distinct superfamily of NAD^+^-dependent deacylases, target substrates through ADPr [52,53].

Based on the number of ADP-ribose units, single or multiple, transferred onto target substrates, ARTs can be further referred to as MARylating or PARylating enzymes, respectively [54].

The human genome encodes for seventeen ARTDs and five ARTCs, the former being also largely termed PARPs [12].Very recently, the nomenclature of ARTD enzymes has been reviewed with the aim to broadly support the scientific community in discussing ADPr biology [55]. In the following sections, the updated nomenclature of mammalian ARTDs will be adopted, accordingly.

All the seventeen members of human ARTD family share a conserved catalytic domain though with limited sequence similarity [35]. Based on their catalytic triad composition, ARTDs are classified into five groups (Table 1).

The first group contains all the enzymes harboring the H-Y-E catalytic triad sequence, such as PARP1-4, Tankyrase1 and Tankyrase2. With the exception of PARP3 and PARP4 that MARylate substrates, the remaining members are considered true PARylating enzymes. Indeed, PARP1 and PARP2 are able to synthesize long linear PAR chains or alternatively branched segments of PAR [70]. Tankyrase1 and Tankyrase2, instead, modify their substrates by the addition of a few units of ADP-ribose to produce short PAR chains of about 20 units in length [70].

The second group harboring the H-Y-I catalytic triad sequence encloses PARP6-8 and PARP10-12; the H-Y-Y catalytic triad sequence is found in PARP16; the H-Y-L is found in PARP14 and PARP15; the Q-Y-T/Y-Y-T are, respectively, found in PARP9 and PARP13. Furthermore, a divergent PARP-like enzyme referred to as TpT1 or KptA, namely ARTD18 [84], contains the triad H-H-V.

Due to technology advancements in the last decade, it has become clear that the majority of ARTDs, i.e., PARP3, PARP4, and PARP6–PARP16, catalyze MARylation reaction with the exception of PARP13, which has been reported to be inactive in agreement to the lack of a conserved catalytic triad composition [12,85]. Recent structural studies on PARP13 show that, in addition to the zinc-finger domains, the WWE-domains are necessary to potentiate PARP13 antiviral activity, supporting the interaction with PAR [86]. The main features of human ARTDs are reported in Table 1.

ARTCs mostly comprise extracellular membrane-bound enzymes, with the exception of ARTC5 that is a secreted enzyme [87]. They have been shown to MARylate substrates both extracellularly and intracellularly, thus regulating cellular functions, including stress response [87,88].

### 3.2. ADP-Ribosylhydrolases

As with other PTMs, ADPr signaling is tightly regulated in the cell by the activity of specialized ADP-ribose processing enzymes known as ADP-ribosylhydrolases. DraG-like ADP-ribosyl-acceptor hydrolases (ARHs) and macrodomain-containing enzymes represent the ADP-ribosylhydrolases known to support this function in humans. Indeed, the human genome encodes three DraG-related ARHs (ARH1, ARH2, and ARH3) involved in the regulation of stress response [89,90,91]. Notably, ARH1 reverses the arginine-ADP-ribosylated substrates catalyzed by mammalian ARTCs and ARH3 shows activity toward Ser-ADPr substrates [91], whilst ARH2 appears to be inactive.

**Figure 1:**
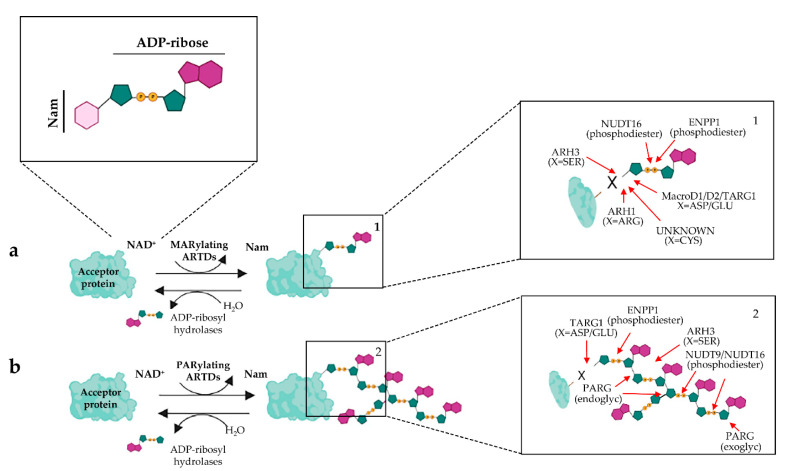
Schematic representation of the ADP-ribosylation reaction onto acceptor protein. (**a**) Mono-ADP-ribosylation reaction is catalyzed by human ARTDs in the presence of the NAD^+^. Inset 1. The modification is reversed by selective ADP-ribosylhydrolases (MacroD1, MacroD2, TARG1, ARH1, ARH3) that display different amino acid–ADP-ribose linkage specificity, and by phosphodiesterases (NUDT16 and ENPP1). (**b**) Poly-ADP-ribosylation reaction is catalyzed by human ARTDs. Inset 2. Linear or branched chains of poly-ADP-ribose are hydrolyzed by selective ADP-riboshydrolases (PARG, ARH3, TARG1) and by phosphodiesterases (NUDT16 and ENPP1). Further details are reported in the text. ARH1/ARH3, ADP-ribosyl acceptor hydrolases 1/3; NUDT16, nudix hydroxylase 16; PARG, poly-ADP-ribosyl glycohydrolase (endo-glyc, endo-glycolytic activity; exo-glyc, exo-glycolitic activity); TARG1, Terminal ADP-ribose glycosylhydrolase 1.

Macrodomain-containing enzymes harbor a common ADP-ribose binding domain, known as macrodomain, and as said above are widely distributed in all domains of life [45,92,93]. These protein modules are able to recognize ADP-ribose, PAR or O-acetyl-ADP-ribose and have been found in the poly-ADP-ribosyl glycohydrolase (PARG), MacroD1, MacroD2 and Terminal ADP-ribose glycosylhydrolase 1 (TARG1) macrodomain-containing enzymes [45,92,94,95]. PARG displays an exo- or endo-glycosyl hydrolytic activity that results in an efficient cleavage of PAR chains, though it is unable to remove the terminal ADP-ribose attached to protein substrates [94]. Conversely, the remaining macrodomain-containing hydrolase, namely TARG1, MacroD1, and MacroD2, are able to remove the ADP-ribose from MARylated substrates [44,96,97,98]. In addition, the enzymes NUDT16 and ENPP1, respectively belonging to the nucleoside diphosphate-linked moiety X (Nudix) and nucleotide pyrophosphatase/phosphodiesterases (NPP) families, are able to hydrolyse the phosphodiester bond within ADP-ribosylated substrates [99,100]. Figure 1 shows the counterplay of ADPr removal.

### 3.3. PAR-Binding Proteins

ADPr signaling is decoded by a multitude of proteins known as PAR-binding proteins that are able to read the ADP-ribosylation modification due to the presence of conserved protein motifs and domains. PAR-binding motifs and domains, namely PAR binding motif (PBM), Poly-ADP-ribose-binding zinc-finger (PBZ) domain, Tryptophan-tryptophan-glutamate (WWE) domain, Oligonucleotide/oligosaccharide-binding fold domain (OB fold), RNA recognition motif (RRM), Arginine-glycine-glycine motif (RGG), PilT N-terminus (PIN) and WD40 domains, have been proven to bind protein-bound or free PAR [45,101]. As a result of this binding, different cellular functions are promptly reprogrammed, such as in DDR, cell cycle, chromatin structure remodeling, stress granule assembly and transcription [102,103,104,105].

### 3.4. ADPr of Macromolecules

The field of ADPr has been enormously expanded in the last ten years and many new findings keep on coming to the forefront. Target identification has always represented a challenging aim in the field. The complexity of targets, partners and biological functions has also paved the way for the set-up of novel technologies and techniques to gain insight into the cellular functions of each ARTD enzyme [102]. Accordingly, in the last five years, the number of ADP-ribosylated substrates has grown largely, together with the fundamental role exerted by MARylation in regulating cell functions [105]. Indeed, initially identified as a protein PTM, ADPr has also been detected on DNA and RNA in mammals as well, widespread in all domains of life [13,106,107,108,109]. Though the biological functions still remain unknown in humans, these findings are suggestive of the fundamental role played by ADPr signaling in the fields of epigenetics and DNA repair [110].

Concerning proteins, mammalian ARTDs target a multitude of substrates at specific amino acidic residues, including serine and tyrosine [111,112,113], negatively charged residues, such as glutamic and aspartic amino acids [114,115,116,117], positively charged lysine [57], glycine as in the case of PARP9 of ubiquitin [118], and cysteine as reported for PARP7 [119,120] and PARP8 [70,121]. However, the biological significance of these modified residues is just starting to emerge, since recent proteomic analyses show that several residues, as serine for instance, are mainly modified in response to genotoxic *stimuli* but not under unperturbed conditions [122]. By contrast, arginine has been identified as the main aminoacidic acceptor, suggesting an evident contribution of ARTC1 regulating functions related to Endoplasmic reticulum and the Golgi apparatus at the basal level. Cysteine residues, instead, have been identified as ADPr acceptor sites in auto-modified PARP8 under physiological conditions, though the role still remains unclear [122].

## 4. The Role of ADPr in RNA Biology

Reversible ADPr reaction regulates a plethora of fundamental biological processes in mammals, as diverse as DDR [26,122,123,124,125,126], transcription, cell division [62,64,127], cell proliferation [128,129,130], cell death [131,132,133,134] immune and stress responses [76,77], as well as other emerging pathways [78,135,136]. For further details on the functional outcomes modulated by ADPr, we refer the reader to the valuable and specialized reviews in the field [24,26,137,138,139,140,141,142]. Furthermore, the imbalance between the addition and the removal of ADP-ribose from substrates plays also a role in different pathological processes, as diverse as neurological disorders [96], tumorigenesis and tumor progression [83], as well as bacterial- and viral-mediated infections [30,69,137,143,144,145,146,147,148].

In the section below, we focus on the role that ADPr signaling exerts in post-transcriptional regulation, modulating different steps of RNA biology at molecular level. Indeed, RNA biology represents an expanding topic of research in the field over the last ten years, where the modification acts as key player in controlling important steps in RNA maturation.

### 4.1. ADPr-Mediated Regulation of Nuclear RNA Processing

Gene expression is a highly regulated biological process that leads to the synthesis of new RNA molecules for the modulation of different functional outcomes. It can be driven in response to intrinsic and extrinsic *stimuli* with the aim to support cell proliferation, survival, or death. Gene expression occurs through several steps that foresee: (i) chromatin modulation; (ii) transcription; (iii) co-regulation; (iv) DNA methylation; and (v) RNA regulation. Early studies in the field were centered on the role of PARP1 and PAR as regulators of the first steps of the process that is tackled by valuable reviews elsewhere [58].

Concerning RNA regulation processes, they enclose: (i) mRNA maturation in the nucleus; (ii) shuttling of mature mRNA to the cytoplasm; (iii) translation into protein; (iv) degradation (mRNA decay); (v) silencing by microRNA (miRNA). All these steps are modulated by a cluster of proteins, namely the RNA-binding proteins (RBPs), whose activity can be affected by PTMs that modulate their interaction with specific RNAs and and/or proteins with effects on processing, localization, translation or stability [149].

Several ARTDs are known to regulate this process, either through direct modification of substrates or by a non-covalent interaction with PAR, thereby leading in both cases to a gain or a loss of function as discussed in detail below.

#### 4.1.1. RNA Regulation at the Nucleus

Nuclear mRNA regulation relies on three steps namely capping, splicing and poly-Adenylation of primary RNA transcripts [150]. Early studies in the field demonstrated the involvement of PARP1 in RNA maturation with implications on the functional role of a sub-group of RBPs, namely heterogeneous nuclear-ribonucleoproteins (hn-RNPs), which are key players in multiple steps of RNA maturation [151]. Indeed, proteomic approaches [152] have demonstrated that many hn-RNPs are PAR-binding proteins, as in the case of hn-RNP A1 that binds poly-ADP-ribose deriving from PARP1 auto-modification. This finding strongly suggested a link between ADPr activity and altered mRNA trafficking. Similarly, Ji and Tulin (2013) [153] have shown that hn-RNPs in *Drosophila melanogaster* are also modulated by ADP-ribosylation, and that PAR-binding causes the dissociation of hnRNPs from pre-mRNA, thus impairing the intron splicing step [153]. Additionally, the regulatory role of ADPr signaling in alternative splicing is also provided by the alternative splicing factor/splicing factor 2 (ASF/FS2) factor, whose binding to PAR prevents its phosphorylation from DNA Topoisomerase. As ASF/FS2 phosphorylation triggers its splicing activity, it is likely that PAR-binding modulates alternative splicing either inhibiting phosphorylation or by hampering the ASF/FS2 interaction with mRNAs [154]. Likewise, other splicing factors, such as SF3B1, SF3A1 and SF3B2, have been found to be linked with PAR [155].

Additionally, upon heat shock stress, PARP1 has been found to modulate poly-Adenylation, the final step before mature mRNA leaves the nucleus to reach the cytoplasm [156]. Under this condition, Poly(A) polymerase (PAP), the enzyme responsible for poly-Adenylation, becomes PARylated losing its polymerase activity, with the resulting blockade of mRNA maturation synthesis.

PARP1-dependent ADPr also affects a group of RNA-binding proteins (RBPs) involved in the nuclear/cytoplasm export of mRNA molecules. Within this group; embryonic lethal abnormal vision-like 1/Hu antigen R (ELAVL1/HuR), a stabilizing mRNA protein able to recognize the AU-rich elements (ARES) at 3′UTR of mRNA, has been shown to interact with PARP1 after lipopolysaccharide (LPS) treatment. Following LPS administration to cells, ELAVL1/HuR results to be poly-ADP-ribosylated and the pharmacological inhibition of PARP1 with PJ34 strongly decreases ELAVL1/HuR shuttling to the cytoplasm [157]. As a result, an increase in ELAVL1/HuR cytosolic localization is observed, together with ELAVL1/HuR mRNA target stability [157]. Collectively, these data highlight the positively regulatory function of PARylation in post-transcriptional regulation in response to inflammatory *stimuli*.

A recent study has further gained insight into this finding underlining the molecular mechanism promoting ELAVL1/HuR translocation [158]. Indeed, PARylation promotes multimeric organization of ELAVL1/HuR that stably interacts with mRNA targets. At the same time, the oligomerization allows the dissociation of the miRNA-induced silencing complex (miRISC) from the target RNA, thus resulting in the stabilization of ARE-containing mRNAs.

Recently, the finding of a novel and selective PARP11 inhibitor, 8-methyl-7-(prop-1-yn-1-yl)-2-((pyrimidin-2-ylthio)methyl)quinazolin-4(3H)-one (ITK7), has also suggested a role for PARP11 at the nuclear envelope [159]. Indeed, HeLa cells overexpressing GFP-PARP11 show a strong reduction in the signal corresponding to mono-ADP-ribosylation around the nuclear envelope after treatment with ITK7, with respect to control cells. This finding is suggestive of a role at nuclear compartment for PARP11 that results to be strictly dependent on catalytic activity.

#### 4.1.2. rRNA at the Nucleolus

Under normal cellular homeostasis, the majority of PARP1 and nuclear PAR are localized in the nucleolus, where they are supposed to be involved in the regulation of rRNA processing and ribosome biogenesis. In *D. melanogaster*, the PARP1 depletion or chemical inhibition of PARP1 has been found to correlate with nucleolus disassembly, storage of rRNA intermediates and decreased polysome formation in the cytoplasm [160], thus strongly suggesting the regulatory role of PARP1 in nucleolar structure maintenance and rRNA processing. Furthermore, following PARP1 inhibition, several nucleolar resident proteins, including coilin and fibrillarin, have been shown to be mislocalized in the cytoplasm, suggesting a role for PARP1 in the regulation of rRNA splicing processes [161].

PARP1 also regulates rRNA transcription through binding to TTF-1-interacting protein 5 (TIP5), a component of the nucleolar remodeling complex (NoRC) [161]. PARP1 is activated by the interaction with the non-coding RNA (pRNA), which promotes the formation of PARP1-pRNA-TIP5 complex. As result, active PARP1 ADP-ribosylates itself, TIP5 and histones, hence leading to transcriptional repression. 

### 4.2. ADPr-Mediated Regulation of RNA Processing at the Cytoplasm

Several steps of the mRNA maturation process are affected by ADPr modification in the cytoplasm, similarly to what occurs in the nucleus. Of note, a group of ARTDs are also identified as RNA-binding proteins due to the presence either of conserved CCCH RNA-binding domains (PARP7, PARP10, PARP11, PARP12 and PARP13) or of RRM motifs (PARP10 and PARP14) [162]. Their role in RNA biology is discussed in the following section.

Once mRNA transcripts reach the cytoplasm, they can undergo protein translation, can be silenced by micro-RNA (miRNA)-mediated post-transcriptional repression, or can be degraded by mRNA decay.

Referring to translation, the primary step, namely initiation, generally occurs by the recognition of 5′CAP modification at the mRNA molecule, even if it can also be achieved through the binding of RBPs to Internal Ribosome Entry Sites (IRES) present on target mRNAs. Inhibition of the interaction between RBPs and IRES elements on target mRNAs halters the translation of the transcript. An example is provided by hnRNA binding protein HRB98DE/HRP38 (HRP38), an RBP in *D. melanogaster*, ortholog of human hnRNPA1, which is described to recruit ribosomes to IRES along the DE-Cadherin mRNA to promote its translation. HRP38 non-covalent binding to PAR hampers binding of ribosomes to IRES region on DE-cadherin mRNA resulting into a block of DE-Cadherin mRNA translation [163]. More recent studies have demonstrated that HRP38 regulates the translation of Nanos mRNA through the binding of its 3′UTR. HRP38 binding to PAR causes a decrease in HRP38 interaction to Nanos 3′UTR, thus leading to an increase in the translation in vivo and in vitro, hence concluding that HRP38 blocks Nanos translation, whereas ADPr relieves the repression effect [163].

A further example of the ADPr regulatory role in translation is provided by Challa and colleagues (2021), who have shown the important role played by PARP16-mediated MARylation of ribosomal proteins in ovarian cancer cells [164]. With the aim to analyze the contribution of NAD^+^ signaling in cancer cells, this study has revealed that PARP16 supports the ADPr of ribosomal protein utilizing NAD^+^ synthesized in the cytoplasm by NAMNT-2, whose protein level is upregulated here. As a result, cytoplasmic increase in mono-ADP-ribose has been demonstrated to correlate with an impairment of polysomes assembly and mRNA loading, thus inhibiting protein synthesis and maintaining protein homeostasis. It followed that under these conditions the ovarian cancer cells were able to grow. Notably, this study proposes a novel therapeutic treatment based on NAMNT-2 depletion or PARP16 inhibition relying on the strategical approach to target the NAD^+^ availability in cancer cells, thereby affecting the downstream NAD^+^-consuming enzymatic pathways.

The impairment of translation in an ADPr-dependent manner is also described as anti-viral mechanism against alphaviruses [68]. The RNA-binding ARTDs PARP7, PARP10 and PARP12 have been shown to strongly induce the clearance of Venezuelan equine encephalitis virus mutants and other alphavirus from infected cells through the regulation of cellular translation and virus replication [69]. In particular, the three enzymes, whose gene expression is upregulated in response to interferon, mediate the antiviral response. The PARP12 long isoform (PARP12L) antiviral activity relies on its own ADPr activity and also on the ability to bind to different interactors in the cytoplasm [69]. The presence of a fully active catalytic domain is required for the efficacious anti-viral response that relies upon the host translational repression, though the underlining molecular mechanism still remains unknown. For more details, we refer the reader to valuable reviews in the ADPr-mediated host–virus interaction field [165,166].

Furthermore, under stressful conditions, several ARTD family members have been reported to regulate gene expression at a post-transcriptional level. Indeed, under oxidative stress, the enzymes PARP12, PARP13.1, PARP13.2, PARP14, PARP15, Tankyrase1 and two isoforms of PARGs assemble in the presence of PAR polymer in the cytoplasm, forming particular structures known as stress granules [24,76,167]. These transient structures also contain the four miRNA-binding Argonaute proteins that result to be poly-ADP-ribosylated upon stress. Consequently, a relief of miRNA-mediated translational repression occurs due to the ADPr-mediated function.

mRNA decay is another ADPr-regulated functional process. PARP13, which is known as inactive ARTD family member, has been described to target cellular mRNAs following the interaction with mRNA transcripts due to the presence of its zinc-finger domains [79,168,169]. The knocking down of PARP13 determines a mis-regulation of the transcriptome with evident effects on mRNAs containing signal sequences for sorting to endoplasmic reticulum, the subcellular compartment where PARP13 is located. Among these transcripts, tumor necrosis factor (TNF)-related apoptosis-inducing ligand receptor 4 (TRAILR4) mRNA is mainly affected, since the PARP13 binding to 3′UTR allows TRAILR4 mRNA degradation through the exosomal pathway [80]. Thus, through the modulation of PARP13, cells can escape the cell death avoiding the accumulation of elevated levels of TRAIL4 mRNA.

A further example is provided by PARP14, a RNA-binding ARTD that is also included in the subclass of the macrodomain-containing-PARPs (MACRO-PARPs) [162]. PARP14 is selectively involved in the post-transcriptional regulation of Tissue factor (TF) (CD142), a key mediator of thrombosis and inflammation, in macrophages [82]. The mechanism relies on the control of TF mRNA stability after the formation of a complex where PARP14 and the mRNA-destabilizing protein tristetraprolin (TTP) interact through the adenylate-uridylate-rich element in the TF mRNA 3′UTR, thus providing a novel insight for the post-transcriptional regulation of TF expression. However, the molecular mechanism underlining this finding remains to be further characterized.

The regulatory role of PARP14 in RNA biology has been further corroborated by the following studies aimed at identifying protein substrates by combining chemical genetics with proximity-dependent labelling. This approach led Carter-O’Connell and colleagues (2018) to identify 114 substrates selectively modified by PARP14, several of which were RNA regulatory proteins [170]. Notably, PARP14 was shown to interact with DEAD-Box Helicase 6 (DDX6) at processing-bodies (P-bodies), membrane-less ribonucleoprotein (RNP) granules, composed of translationally repressed mRNAs and proteins related to mRNA decay, thereby suggesting a role in post-transcriptional regulation. Intriguingly, PARP13, known to play a role in regulating mRNA stability (see above), has been found ADP-ribosylated by PARP14 at different fourteen amino acidic sites, leading to speculate on the possible crosstalk between the different members of ARTD subfamily within the RNA biology process.

Collectively, these data add new layers of complexity to ADPr signaling.

## 5. Targeting ADPr Signaling for Therapeutic Treatment

### 5.1. Chemical Inhibition of ARTDs

Small-compound inhibitors have been fundamental tools to unveil ARTDs’ biological functions. In addition, the pivotal role played by ADPr in regulating pathophysiological processes has fostered the search for new molecules to be exploited as inhibitors for therapeutic treatment development.

Pioneering studies focused on the ARTD enzymes that carry out PARylation with implications in genome integrity and signal transduction. Thus, it followed that targeting DDR could represent a promising strategy to counteract cancer cells. PARP1 small-molecule inhibitors, designed as NAD^+^ analogs, induce conformational changes in PARP1 and stabilize the complex between PARP1 and DNA, thereby “trapping” the complexes at DNA lesions. Therefore, PARP1 inhibitors have been applied as anticancer agents relying on the strategical induction of “synthetic lethality” in cancer cells carrying Breast-Cancer 1/2 (*BRCA1/2)* mutations [171,172]. PARP-inhibitors approved by the U.S. Food and Drug Administration (FDA), namely niraparib, olaparib, talazoparib, and rucaparib, are currently used in the therapeutic treatment of BRCA-mutated breast and ovarian cancers, as well as in tumors with dysfunctional *BRCA* genes [173]. The range of treated cancers and the uses of PARP inhibitors may increase as more research is completed.

Intriguingly, a more recent approach aims at harnessing these inhibitors in the treatment of non-oncological diseases, including oxidative stress, inflammatory responses, as well as neurodegenerative and cardiovascular diseases [137,174,175].

There is a marked over-activation of PARP1 in the reperfused myocardium, which parallels with the decline of the contractile function and myocardial NAD^+^ and ATP contents in preclinical models of myocardial infarction and cardiopulmonary bypass. The pharmacological inhibition of PARP with various inhibitors or its genetic deletion, markedly improves the outcome of myocardial ischemia-reperfusion damage (in all in vitro or ex vivo models), which is also a subject of numerous overviews [176]. Aside from the DNA damage repair, other ADPr-dependent molecular pathways have been evaluated as therapeutic targets, including those in which hydrolases revert the reaction. We direct the reader to recent valuable reviews providing a comprehensive state of the art on this topic [177,178].

Since one concern is always the specificity, it is worth noting that for niraparib the results from the last phase III trial “support the hypothesis that niraparib has mechanisms of action other than those involved in the repair of DNA damage”, as the Authors note. These could include PARP-regulated gene transcription, ribosome biogenesis and immune activation, they speculate [179].

The mitotic checkpoint protein CHFR (Checkpoint With Forkhead And Ring Finger Domains) has emerged as a major mediator of taxane resistance in cancer. It has been shown that CHFR’s PAR-binding zinc finger domain (PBZ) mediates a protein interaction with poly-ADP-ribosylated PARP1 leading to the stabilization of CHFR. The disruption of the CHFR-PARP1 interaction through a PBZ domain peptide induces the loss of CHFR protein expression. These findings provide a proof-of-principle that the small molecule inhibition of the CHFR-PBZ domain interaction can be exploited to increase the efficacy of taxane-based chemotherapy in cancer [180,181].

Therefore, compounds that are capable of disrupting the protein–protein interactions of PARP1 provide an alternative by inhibiting its activities with improved selectivity profiles. 

Another major limitation depends on the shortage of novel compounds to inhibit selectively all the enzymes of the ARTD family and the research in this field is still ongoing [173]. The latest findings on the role of MARylating enzymes highlight the need of new inhibitors to unravel and dissect the emerging signaling pathways in which mono-ADP-ribosylation plays a role.

As such, the finding of several ARTDs, other than PARP1, as regulators of RNA biology, may provide alternative strategies to exploit as potential therapeutic targets. Of note, the observation that NAMNT2-dependent NAD^+^ synthesis parallels PARP16 activity in ovarian cancer cells, suggests novel approaches in cancer therapies, which include the enzymes responsible for NAD^+^ biosynthetic pathways as well [169]. The impairment of the crosstalk between signaling pathways and RNA processing is frequently linked to pathologies, such as neurodegenerative diseases and cancer. The current knowledge about the modulation of RNA biology is still at the beginning. The number of RBPs involved is continuously expanding, likewise the role played by different PTMs in modulating their functions, localizations or degradation. As the dysregulation of PTMs in RBPs has been associated with the pathophysiological mechanism of diverse diseases, it is reasonable to consider the ADP-ribosylating enzymes involved in the modulation of RNA processing as good candidates for the assessment of novel therapeutic options. 

Novel strategies have to be addressed for the development of new classes of ARTD inhibitors evaluating the potential of binding sites and motifs other than catalytic sites [173,177].

Last but not least, the post-transcriptional regulation of ARTD-encoding mRNAs may represent a further strategy to take into consideration. Indeed, PARP1 expression is regulated by the nuclear factor 90 (NF90), a RNA binding protein able to bind and stabilize PARP1 mRNA [182]. Targeting this interaction and therefore mRNA stability might be assessed as alternative therapeutic intervention as well.

### 5.2. New Avenues: The Post-Transcriptional Regulation of ARTDs mRNA

RBPs have been shown to control the expression of many genes by binding to the respective mRNA species, encoding proto-oncogenes, growth factors, cytokines, transcription factors, and other proteins in various cell types [183].

The 3′UTRs and the 5′UTRs are the transcript target sequences involved in the RBPs binding and mediating the formation of the so-called “RNA operon”. This is a functional unit in which multiple physiologically related transcripts are coordinately regulated during splicing, export, stability, localization, and translation. These subpopulations of mRNAs bind the same RBPs in a dynamic manner because each mRNA can join different “RNA operons” [184,185,186].

As a proof of concept, we have recently reported about the “RNA operon” regulatory mechanism for the human Major Histocompatibility Complex II gene [187] and for the human Paraoxonase 2 (PON2) [117].

The modulation of mRNA stability is now considered as a novel therapeutic approach together with other functions related to the RNA biology [188,189,190].

The competition of miRNAs and antisense oligonucleotides (ASO) with RBPs for binding to the same RNA binding site or the modification of RBPs affinity via PTMs, proteins or small molecules interactions are now commonly used [149,191].

Post-transcriptional gene regulation via RBPs is an adaptable reprograming mechanism that may drive PARP inhibitor resistance. Chand and colleagues (2017) have previously shown that the RBP ELAVL1/HuR promotes a drug-resistant phenotype, through its stress-induced cytoplasmic translocation and stabilization of pro-survival mRNA targets, among which PARG [192]. The role of ELAVL1/HuR is further discussed in Section 4.1.1.

In the vein of this research, we recently analyzed a database of human genes categorized on the basis of conserved short sequences (n-mers) at the 3′UTR [183]. The Authors demonstrated how the database provides evidence that these n-mers are potentially involved in regulatory functions. The identified n-mers in fact overlap with previously recognised binding sites for ELAVL1/HuR and T-Cell-Restricted Intracellular Antigen-1 (TIA-1) and with ARE and GU-rich element (GRE) sequences. It was established also that n-mers overlap with predicted miRNA target sites. Finally, a method to cluster n-mer groups allowed the identification of putative gene networks. This analysis indicated that several sets of gene clusters share commonalities in terms of pathway and function. Moreover, interacting proteins turned out to be present in numerous clusters [183].

Based on this research, we decided to explore the clusters containing the genes for ARTDs proteins.

As reported in Table 2, among the seventeen *ARTDs* no clusters were found for *PARPs* 1–4, 5b, 6, 7, 10, and 13.

For the *ARTDs* involved in the clusters, their number was quite different, ranging from 1 (*PARP8* and *15*) to 20 for *PARP11*. In addition, the number of genes in each cluster was quite various, ranging from a minimum of 20 to a maximum of 125. Given that all the genes belonging to a single cluster share a conserved sequence at the 3′UTR, this leads to assess the possibility that their transcripts can be co-regulated at a post-transcriptional level.

As an example, the regions conserved at the 3′UTR of the two *PARP12* clusters are the following: AGUUUUAGUUUU (cluster 1) and UAAUGAUUUUU (cluster 2).

Regarding the cluster 1 signature, this is closely related to the hsa-mir-3925 precursor miRNA found using the mirBase program (https://www.mirbase.org/search-rnacentral.shtml, accessed on 24 February 2022).

This sequence is part of the miRNA stem and by the binding of a hypothetical RBP there might be an interference in the Dicer processing. The gene *DICER1*, detected in cluster 2, encodes a ribonuclease that trims double stranded RNA or pre-miRNA to form small interfering RNA or microRNA, respectively. These processed RNAs are incorporated into the RNA-induced silencing complex (RISC), which targets messenger RNA to prevent translation. This specific miRNA has been related to cancer in females [131,193].

**Figure 2:**
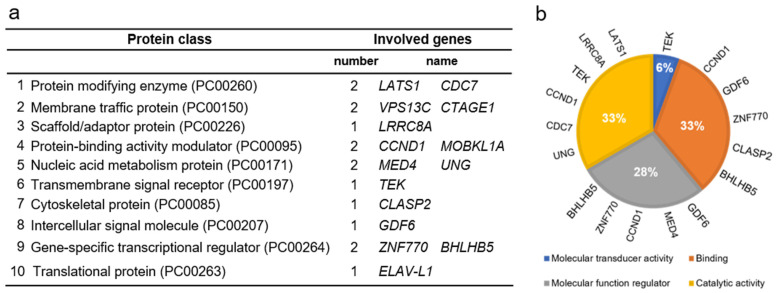
Representation of the GO analysis using the 21 genes list of Cluster 2 as input (Table S1 [194, 197,198,199,200,201,202,203,204,205,206,207,208,209,210,211,212,213,214,215,216,217,218]): (**a**) categorization by protein class; (**b**) grouping by molecular functions. Six genes, namely *PARP12, DICER1, LUZP4, UPF3B, ZFAND3,* and *OSGIN2*, are not included.

Supplementary Table S1 provides the list of the 21 genes related to cluster 2 with the indicated function as reported in the Genecard database (https://www.genecards.org/, accessed on 28 January 2022). The genes have been further categorized by the Gene Ontology analysis using Panther Classification system (http://pantherdb.org/, accessed on 28 January 2022) and reported in Figure 2.

Out of the 21 genes of cluster 2, 13 were grouped according to 4 molecular functions (Figure 2b), whereas 15 according to 10 specific protein classes (Figure 2a). The remaining 6 genes are RBPs and DNA binding proteins that are not classified, likely, because of being part of many pathways.

Intriguingly, this cluster contains, among other genes, *DICER1*, which acts as a strong antiviral agent with activity against RNA viruses, including the Zika and SARS-CoV-2 viruses. This activity resembles the above-described antiviral role of PARP12 and PARP13. The protein encoded by ELAVL1/HuR gene as said above is a member of the ELAVL family of RBPs that binds AU-rich elements (AREs) found at the 3′UTR of many mRNAs. AREs by binding to RBPs or miRNA signal the degradation/stabilization of mRNAs as a means to regulate gene expression. The ELAVL family of proteins in general plays a role in stabilizing ARE-containing mRNAs. This gene family has been implicated in a variety of biological processes and has been linked to a number of diseases, including cancer [194]. ELAVLl binds to the AU-rich element in FOS and nterleukin-3 (IL3) mRNAs. In the case of the FOS AU-rich element, it binds to a core element of 27 nucleotides that contain AUUUA, AUUUUA, and AUUUUUA motifs. These motifs are reminiscent of the two conserved sequences reported above. ELAVL1 is also known to be PARylated by PARP1 in response to LPS exposure. That increases ELAVL1/HuR nucleo-plasmatic translocation and mRNA stabilization, affecting downstream regulation [157]. We are tempted to speculate that PARP12 is able to PARylate ELAVL1/HuR as well, and that both are co-regulated. In agreement with this idea, it has been reported: (i) the elevated expression in HuR-double-transgenic Traffic/HuR Pdx1/Cre mice (TC mice) of two ARTDs, PARP12 and PARP14 [195]; (ii) the physical interaction between ELAVL1/HuR and PARP12 [196].

Additional RBPs contained in the cluster are LUZP4 and UPF3B. LUPZP4 is involved in mRNA export from the nucleus. UPF3B is involved in the regulation of nonsense mediated mRNA Decay (Table S1 [194, 197,198,199,200,201,202,203,204,205,206,207,208,209,210,211,212,213,214,215,216,217,218]). Experimental studies based on RBP depletion by RNA interfering, measuring of gene expression by real-time PCR, immunoprecipitation of RBP(s) bound mRNA and sequencing after PCR amplification, in situ RNA-labelled probing of SDS-PAGE separated proteins followed by identification by MS, analysis of gene relationships by many different algorithms are needed to prove the interactions among members of the cluster and with the relevant PARP.

## 6. Conclusions

Gene expression programs support the cell in division, differentiation, as well as in adaptation to intrinsic and extrinsic *stimuli* or fate decision. The maintenance of cell homeostasis is gained through well inter-connected regulatory processes that are finely tuned by PTMs. In this paper, the regulatory role of ADP-ribosylation in RNA biology has been reviewed, highlighting the RNA processes known to date that are under its control. Indeed, protein substrate modification or non-covalent interaction with PAR induce a range of effects that lead to enzyme inactivation, change in subcellular localization, or stall in the assembly of intracellular organelles with the consequent alteration, loss or gain, of functions as discussed above. However, novel functional processes related to RNA processing regulation are expected to emerge in the near future as an improvement of alternative approaches, as we propose by dealing with the “RNA operon” approach. In addition, the discovery that few of the ARTD family members (PARP10, PARP11 and PARP15) are capable of modifying RNA in vitro adds another layer of complexity in the modulation of RNA biology. Further efforts have to be directed at elucidating the functional role of RNA ADP-ribosylation in cells.

In the last decade, the understanding of ADPr biology has increased substantially. The development of new and more specific technologies has pushed the knowledge in the field enormously, with particular emphasis on MARylation reaction, leading to the identification of novel targets and the discovery of unknown biological functions. In addition, several ARTs have been shown to be involved in the pathogenesis of human diseases, thus evidencing the need for novel specific inhibitors to address ARTs pharmacological targeting. In this review, we propose an alternative approach to analyze the post-transcriptional regulation of ARTs in order to provide new insights into the finding of novel drug targets.

ADPr is important for many fundamental biological processes. However, a complete inventory of modification sites, also depending on changing of specificity, has not been established.

Furthermore, although PTM coverage by MS-based methods is impressive, it still needs to be improved, especially in tissues and in clinically relevant systems.

Regulatory sequences, either placed at promoter regions or on UTRs, function as targets recognized by regulators that can then activate or repress different groups of genes according to the necessity. While regulatory sequences involved in transcription are quite well documented, there is a lack of information on sequence elements involved in post-transcriptional regulation. Here, we reconsidered an already published study, by highlighting conserved sequences in clusters of genes containing those coding for ADP-ribosyltransferase enzymes.

As reported for PARP12 as an example, this analysis can reveal some new relationships among genes that can be useful to understand the functions and propose new opportunities for therapeutic interventions.

By looking at the data of Table 2, there are a lot of clusters that need to be experimentally analyzed, containing around 1900 genes in total. On the other hand, much information is accumulating on the identification of the pathways in which ARTs are involved. The combination of these two areas of research is expected to foster the knowledge on the mechanisms of action of these enzymes and the possibility to use new therapeutic tools.

## Figures and Tables

**Table 1:** Summary of the main features of ARTD family members showing the catalyzed enzymatic activity and the related biological processes in which they are involved.

ARTD	Activity	Catalytic Triad Sequence	ADP-Ribosylated Macromolecules	Key Functions	References
* PARP1	Poly	H-Y-E	Proteins, DNA	DNA damage repair,	[56]
				Chromatin regulation,	[57]
				Gene expression,	[58]
				Immune response	[59]
PARP2	Poly	H-Y-E	Proteins, DNA	DNA damage repair	[60]
PARP3	Mono	H-Y-E	Proteins, DNA	DNA damage repair,	[61]
				mitotic progression	[62]
PARP4	Mono	H-Y-E	Proteins	Vault particle	[63]
TNKS1	Poly/Oligo	H-Y-E	Proteins	Cell division,	[64]
				Genome integrity,	[65]
				Immune response, Protein turnover	[66]
TNKS2	Poly/oligo	H-Y-E	Proteins	Cell division, Genome integrity	[67]
PARP6	Mono	H-Y-I	Proteins	Dendrite	[68]
PARP7	Mono	H-Y-I	Proteins	Immune response	[69]
PARP8	Mono	H-Y-I	Proteins	Cell Viability	[70]
PARP9	Inactive/Mono	Q-Y-T	Proteins	Immune response	[71]
PARP10	Mono	H-Y-I	Proteins, RNA	DNA damage repair,	[72]
				Immune response	[73]
PARP11	Mono	H-Y-I	Proteins, RNA	Nuclear pore function,	[74]
				Immune response	[75]
PARP12	Mono	H-Y-I	Proteins	Stress granule function	[76,77]
				Immune Response	[69]
				Intracellular trafficking	[78]
PARP13	Inactive	Y-Y-T	ND	Immune response	[79,80]
PARP14	Mono	H-Y-L	Proteins	Stress granule function,	[76]
				cytoskeleton regulation	[70]
				Immune response	[81]
				Post-transcriptional gene regulation	[82]
PARP15	Mono	H-Y-L	Proteins, RNA	Stress granule function	[76]
PARP16	Mono	H-Y-Y	Proteins	Unfolded protein response	[83]

* Colors define the different ARTDs groups based on catalytic triad composition: H-Y-E-containing ARTDs in light grey; H-Y-I-containing ARTDs in yellow; H-Y-Y-containing ARTDs in light violet; H-Y-L-containing ARTDs in orange; Q-Y-T-containing ARTD in pink; Y-Y-T-containing ARTD in red.

**Table 2:** Gene cluster database analysis for genes including *PARPs*.

*ARTD* Gene	Total Number of Clusters Containing the *ARTD* Gene	Number of Genes in Each Cluster																			
*PARP5a*	10	34	32	30	28	23	22	22	21	21	21										
*PARP8*	1	23																			
*PARP9*	3	48	21	20																	
*PARP11*	20	125	93	41	40	40	37	32	30	26	26	25	24	24	23	23	22	22	21	21	21
*PARP12*	2	37	21																		
*PARP14*	18	53	50	48	47	47	44	41	41	40	39	35	34	32	30	26	24	24	23		
*PARP15*	1	24																			
*PARP16*	2	33	22																		
Cluster numbering		1	2	3	4	5	6	7	8	9	10	11	12	13	14	15	16	17	18	19	20

## Data Availability

Not applicable.

## Supplementary Materials

The following supporting information can be downloaded at: https://www.mdpi.com/article/10.3390/biom12030443/s1, Table S1: PARP12 cluster of genes with their function (GeneCard).
