# Supplementary material for: ADP-Ribosylation Post-Translational Modification: An Overview with a Focus on RNA Biology and New Pharmacological Perspectives
Source: Biomolecules. 2022 Mar 13;12(3):443. doi: 10.3390/biom12030443 (PMC8946771; doi:10.3390/biom12030443)
Supplement: Supplementary file 1 [file biomolecules-12-00443-s001.zip › biomolecules-1601262-SI.pdf]

biomolecules

MDPI

Review

ADP-Ribosylation Post-Translational Modification: AnOverview with a Focus on RNA Biology and New Pharmacological Perspectives

Giuseppe Manco [1,*], Giuseppina Lacerra [2], Elena Porzio [1] and Giuliana Catara [1,*]

[1] Institute of Biochemistry and Cell Biology, CNR, Via P. Castellino 111, 80131 Naples, Italy; elena.porzio@cnr.it
[2] Institute of Genetics and Biophysics "Adriano Buzzati-Traverso", CNR, Via P. Castellino 111, 80131 Naples, Italy; giuseppina.lacerra@igb.cnr.it
* Correspondence: giuseppe.manco@cnr.it (G.M.); giuliana.catara@cnr.it (G.C.)

Supplementary Table S1. *PARP12* cluster of genes with their function (GeneCard). The genes have been subdiveded in relation to their gene ontology (Panther) based on the functional classification

Gene	Function (Gencard)
	**Binding**
GDF6	This gene encodes a secreted ligand of the TGF-beta (transforming growth factor-beta) superfamily of proteins [197]. Ligands of this family bind various TGF-beta receptors leadingto recruitment and activation of SMAD family transcription factors that regulate gene expression. The encoded preproprotein is proteolytically processed to generate each subunit of the disulfide-linked homodimer. This protein is required for normal formation of some bones and joints in the limbs, skull, and axial skeleton. Mutations in this gene are associated with Klippel-Feil syndrome, microphthalmia, and Leber congenital amaurosis
UPF3B	This gene encodes a protein that is part of a post-splicing multiprotein complex involved in both mRNA nuclear export and mRNA surveillance [198]. The encoded protein is one of twofunctional homologs to yeast Upf3p. mRNA surveillance detects exported mRNAs with truncated open reading frames and initiates nonsense-mediated mRNA decay (NMD). Whentranslation ends upstream from the last exon-exon junction, this triggers NMD to degrade mRNAs containing premature stop codons. This protein binds to the mRNA and remains bound after nuclear export, acting as a nucleocytoplasmic shuttling protein. It forms with Y14a complex that binds specifically 20 nt upstream of exon-exon junctions. This gene is located on the long arm of chromosome X. Two splice variants encoding different isoforms have been found for this gene.
ZNF770	ZNF770 (Zinc Finger Protein 770) is a Protein Coding gene. Among its related pathways are Gene Expression. Gene Ontology (GO) annotations related to this gene include nucleic acid binding and DNA-binding transcription factor activity, RNA polymerase II-specific [199]. An important paralog of this gene is ZSCAN29.
CCND1	The protein encoded by this gene belongs to the highly conserved cyclin family, whose members are characterized by a dramatic periodicity in protein abundance throughout the cellcycle [200]. Cyclins function as regulators of CDK kinases. Different cyclins exhibit distinct expression and degradation patterns which contribute to the temporal coordination of each mitotic event. This cyclin forms a complex with and functions as a regulatory subunit of CDK4or CDK6, whose activity is required for cell cycle G1/S transition. This protein has been shownto interact with tumor suppressor protein Rb and the expression of this gene is regulated positively by Rb. Mutations, amplification and overexpression of this gene, which alters cell cycle progression, are observed frequently in a variety of human cancers.

CLASP2	CLASP2 (Cytoplasmic Linker Associated Protein 2) is a Protein Coding gene [201]. Diseases associated with CLASP2 include Camptodactyly-Arthropathy-Coxa Vara-Pericarditis Syndrome. Among its related pathways are Signaling by Rho GTPases and Cell Cycle, Mitotic.Gene Ontology (GO) annotations related to this gene include binding and microtubule plus-end binding [202].
BHLHB5	This gene encodes a protein that belongs to the basic helix-loop-helix (bHLH) family of transcription factors that regulate cell fate determination, proliferation, and differentiation [203]. A similar protein in mouse is required for the development of the dorsal cochlear nuclei, and is thought to play a role in in the differentiation of neurons involved in sensory input. The mouse protein also functions in retinogenesis.
	**Catalytic activity**
LATS1	The protein encoded by this gene is a putative serine/threonine kinase that localizes to the mitotic apparatus and complexes with cell cycle controller CDC2 kinase in early mitosis [204].The protein is phosphorylated in a cell-cycle dependent manner, with late prophase phosphorylation remaining through metaphase.<br>LATS1 (Large Tumor Suppressor Kinase 1) is a Protein Coding gene. Diseases associated withLATS1 include Malignant Peritoneal Mesothelioma and Tetraploidy. Among its related pathways are DNA Damage and Signaling by GPCR. Gene Ontology (GO) annotations relatedto this gene include transferase activity, transferring phosphorus-containing groups and protein tyrosine kinase activity.
TEK	This gene encodes a receptor that belongs to the protein tyrosine kinase Tie2 family [205]. Theencoded protein possesses a unique extracellular region that contains two immunoglobulin- like domains, three epidermal growth factor (EGF)-like domains and three fibronectin type IIIrepeats. The ligand angiopoietin-1 binds to this receptor and mediates a signaling pathway that functions in embryonic vascular development. Mutations in this gene are associated withinherited venous malformations of the skin and mucous membranes. Alternative splicing results in multiple transcript variants. Additional alternatively spliced transcript variants of this gene have been described, but their full-length nature is not known.
LRRC8A	This gene encodes a protein belonging to the leucine-rich repeat family of proteins, which areinvolved in diverse biological processes, including cell adhesion, cellular trafficking, and hormone-receptor interactions [206]. This family member is a putative four-pass transmembrane protein that plays a role in B cell development. Defects in this gene cause autosomal dominant non-Bruton type agammaglobulinemia, an immunodeficiency disease
	resulting from defects in B cell maturation. Multiple alternatively spliced transcript variants, which encode the same protein, have been identified for this gene.
CCNC1	
CDC7	This gene encodes a cell division cycle protein with kinase activity that is critical for the G1/S transition [207]. Overexpression of this gene product may be associated with neoplastic transformation for some tumors [208].<br>Diseases associated with CDC7 include Fanconi Anemia, Complementation Group D2 and Glaucoma, Primary Open Angle. Among its related pathways are Mitotic G1-G1/S phases andDNA Damage. Gene Ontology (GO) annotations related to this gene include transferase activity, transferring phosphorus-containing groups and protein tyrosine kinase activity.

UNG	This gene encodes one of several uracil-DNA glycosylases [209]. One important function of uracil-DNA glycosylases is to prevent mutagenesis by eliminating uracil from DNA moleculesby cleaving the N-glycosylic bond and initiating the base-excision repair (BER) pathway. Uracil bases occur from cytosine deamination or misincorporation of dUMP residues. Alternative promoter usage and splicing of this gene leads to two different isoforms: the mitochondrial UNG1 and the nuclear UNG2. The UNG2 term was used as a previous symbolfor the CCNO gene (GeneID 10309), which has been confused with this gene, in the literature and some databases.
	**Molecular function regulator**
GDF6	
ZNF770	
CCND1	
MED4	This gene encodes a component of the Mediator complex [210]. The Mediator complexinteracts with DNA-binding gene-specific transcription factors to modulate transcription by RNA polymerase II. Alternatively spliced transcript variants encoding multiple isoforms have been observed for this gene.
BHLHB5	
	**Molecular transducer activity**
TEK	
	**Kinase actvator**
MOBKL1A	The protein encoded by this gene is similar to the yeast Mob1 protein. Yeast Mob1 binds Mps1p, a protein kinase essential for spindle pole body duplication and mitotic checkpoint regulation [211]. Three transcript variants encoding different isoforms have been found for this gene.
	**PARP**
PARP12	Mono-ADP-ribosyltransferase that mediates mono-ADP-ribosylation of target proteins. PARPtransfers ADP-ribose from nicotinamide dinucleotide (NAD) to Glu/Asp residues on the substrate protein [212].
	**RNA regulation**
DICER1	This gene encodes a protein possessing an RNA helicase motif containing a DEXH box in itsamino terminus and an RNA motif in the carboxy terminus [213]. The encoded protein functions as a ribonuclease and is required by the RNA interference and small temporal RNA
	(stRNA) pathways to produce the active small RNA component that represses gene expression. This protein also acts as a strong antiviral agent with activity against RNA viruses,including the Zika and SARS-CoV-2 viruses. Alternative splicing results in multiple transcript variants.
ELAV1	The protein encoded by this gene is a member of the ELAVL family of RNA-binding proteinsthat contain several RNA recognition motifs, and selectively bind AU-rich elements (AREs) found in the 3' untranslated regions of mRNAs [194]. AREs signal degradation of mRNAs as a means to regulate gene expression, thus by binding AREs, the ELAVL family of proteins play a role in stabilizing ARE-containing mRNAs. This gene has been implicated in a variety of biological processes and has been linked to a number of diseases, including cancer. It is highly expressed in many cancers, and could be potentially useful in cancer diagnosis, prognosis, and therapy.
LUZP4	This gene encodes a leucine-zipper protein that was first defined as a cancer testis antigens. The encoded protein is an RNA binding protein that interacts with the mRNA export receptornuclear RNA export factor 2 [214]. Alternate splicing results in multiple transcript variants.
	**Membrane traffic protein**
VPS13C	This gene encodes a member of the vacuolar protein sorting-associated 13 gene family [215].

	Alternate splicing results in multiple transcript variants.
CTAGE1	CTAGE1 (Cutaneous T Cell Lymphoma-Associated Antigen 1) is a Protein Coding gene.Diseases associated with CTAGE1 include Cutaneous T Cell Lymphoma and Lymphoma [216]. An important paralog of this gene is MIA2.
	**Zing Finder Protein**
ZFAND3	ZFAND3 (Zinc Finger AN1-Type Containing 3) is a Protein Coding gene [217]. Diseasesassociated with ZFAND3 include Maturity-Onset Diabetes Of The Young. An important paralog of this gene is ZFAND6.
	**Involved in the Oxidative stress**
OSGIN2	OSGIN2 (Oxidative Stress Induced Growth Inhibitor Family Member 2) is a Protein Codinggene. Diseases associated with OSGIN2 include Nijmegen Breakage Syndrome [218]. An important paralog of this gene is OSGIN1.
